# Mesoporous hollow black TiO_2_ with controlled lattice disorder degrees for highly efficient visible-light-driven photocatalysis†

**DOI:** 10.1039/c9ra08148h

**Published:** 2019-11-12

**Authors:** Xiongrui Jiang, Zhiyao Yan, Jing Zhang, Junzheng Gao, Wanxia Huang, Qiwu Shi, Hengzhong Zhang

**Affiliations:** College of Materials Science and Engineering, Sichuan University Chengdu 610065 Sichuan China shiqiwu@scu.edu.cn huangwanxia@scu.edu.cn; College of Architecture and Environment, Sichuan University Chengdu 610065 Sichuan China; Center for High Pressure Science and Technology Advance Research Shanghai 201203 China

## Abstract

Black TiO_2_ has received tremendous attention because of its lattice disorder-induced reduction in the TiO_2_ bandgap, which yields excellent light absorption and photocatalytic ability. In this report, a highly efficient visible-light-driven black TiO_2_ photocatalyst was synthesized with a mesoporous hollow shell structure. It provided a higher specific surface area, more reaction sites and enhanced visible light absorption capability, which significantly promoted the photocatalytic reaction. Subsequently, the mesoporous hollow black TiO_2_ with different lattice disorder-engineering degrees were designed. The structure disorder in the black TiO_2_ obviously increased with reduction temperature, leading to improved visible light absorption. However, their visible-light-driven photocatalytic efficiency increased first and then decreased. The highest value can be observed for the sample reduced at 350 °C, which was 2-, 1.4- and 5-fold that of the samples reduced at 320 °C, 380 °C and 400 °C, respectively. This contradiction can be ascribed to the varied functions of the surface defects with different concentrations in the black TiO_2_ during the catalytic process. In particular, the defects at low concentrations boost photocatalysis but reverse photocatalysis at high concentrations when they act as charge recombination centers. This study provides significant insight for the fabrication of high-efficiency visible-light-driven catalytic black TiO_2_ and the understanding of its catalysis mechanism.

## Abstract 2

Our work provides significant insights into the design of hollow black TiO_2_ spheres and the mechanism accounting for their high-efficient visible-light-driven catalysis.

## Graphical abstract

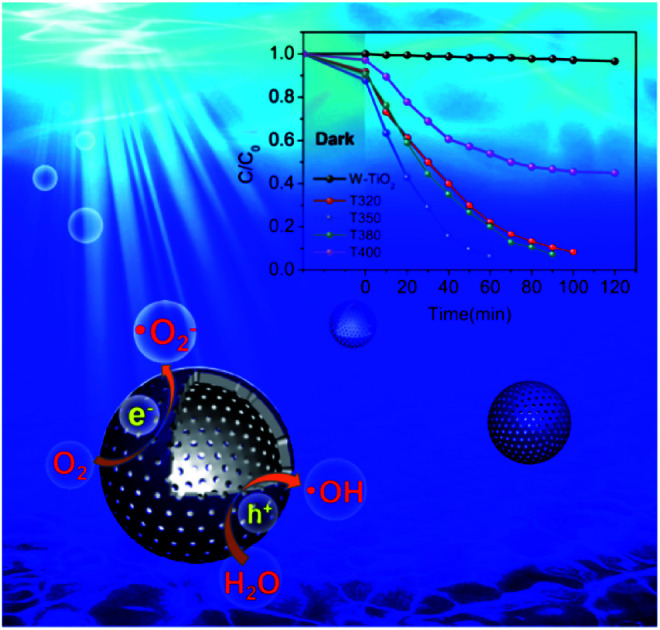

## 1. Introduction

Titanium dioxide, with its high light-harvesting, high chemical stability, low cost, non-toxicity and high resistance to photo corrosion, has been deeply investigated over the years.^1–3^ This has led to its wide potential applications in photocatalysis,^4,5^ self-cleaning coatings,^6,7^ hydrogen generation^8,9^ and photocatalytic sensors,^10,11^*etc.* However, due to its wide bandgap of 3.2 eV, it can work under the ultraviolet band only, which accounts for less than 5% of the entire solar energy.^12^ The poor visible light absorption limits its application in photocatalysis. Therefore, the preparation of high-efficiency TiO_2_ catalysts remains a challenge.

To date, many strategies have been proposed to improve the catalytic ability of TiO_2_. The introduction of impurities or defects is a basic approach, including metal-ion-doping,^13,14^ nonmetal-ion-doping^9,12,15^ and semiconductor recombination.^3,16^ The doping could produce mid-gap states and increase the visible light absorption of TiO_2_, but generally the overall photocatalytic efficiency was still insufficient. Recently, Chen *et al.*^1^ synthesized a new titanium polymorph, black TiO_2_, which was formed by introducing a disordered amorphous layer on the surface of TiO_2_ using hydrogen as a reducing agent; the discolored TiO_2_ exhibited a preferable catalytic effect of five times that before solar light irradiation. Since then, the fabrication of black TiO_2_ and the mechanisms related to its photochemical properties have attracted increasing attention due to the unique visible light absorption characteristics and its great application potential in photocatalysis and hydrogen generation. However, the initial method for the fabrication of black TiO_2_ requires harsh experimental conditions, such as a rigorous reducing atmosphere using H_2_ at high temperatures. Therefore, some other strategies have been developed to seek milder preparation conditions, such as aluminum reduction,^15,17^ plasma treatment,^18,19^ electrochemical reduction–anodization,^20,21^ and solvothermal synthesis.^22,23^ In particular, the solid-state chemical reduction approach to the lattice disorder-engineering of TiO_2_ using NaBH_4_ (ref. 24 and 25) as the reducing agent works at mild temperatures (300–350 °C), which makes it more practical and suitable for large-scale production. Moreover, such low temperatures make it more convenient to design some special nanostructures in black TiO_2_, such as nanorods.^26^ It should be noted that the microstructure design of TiO_2_ has been widely regarded as an efficient method for improving catalytic ability. For example, surface engineering is a vital tool for enhancing the high photocatalytic ability;^27^ mesoporous TiO_2_ (ref. 28) and self-supported TiO_2_ hollow spheres^29^ also boost the photocatalysis efficiency. Nevertheless, very few studies on the morphology design of black TiO_2_ have been reported.

Most of the previous studies focused on the design of black TiO_2_ with high visible-light absorption and highly efficient visible-light catalytic ability.^17,23^ Nevertheless, several recent discoveries have indicated that the visible-light catalytic ability of black TiO_2_ is not dependent on its visible-light absorption. For example, the sample with the highest photoabsorption did not have the most efficient photocatalytic properties.^24,30^ As such, the catalytic mechanism in black TiO_2_ with a disordered structure needs further exploration.

Herein, a mesoporous black TiO_2_ hollow sphere with large specific surface area was prepared *via* a facile evaporation-induced self-assembly (EISA) method. We designed black TiO_2_ hollow spheres with different lattice disorder-engineering degrees and elucidated the contradiction between their visible light absorption and photocatalytic ability. The sample with a suitable concentration of surface defects, combined with the special mesoporous hollow structure, demonstrated a significant improvement in its visible light catalytic ability. In this study, we report the synthesis of a novel nanostructured black TiO_2_ and propose an improved photocatalytic mechanism for the development of black TiO_2_ with enhanced visible-light-driven photocatalytic properties.

## 2. Experimental section

### 2.1 Materials

Tetraethyl orthosilicate (TEOS, 99%) was purchased from Shanghai http://Aladdin-e.com, China. Absolute ethanol (EtOH), sodium hydroxide (NaOH), ammonia solution (NH_4_OH), and titanium(iv) tetrabutoxide (TBOT) were purchased from Chengdu Kelong Reagent Company, China. Polyvinylpyrrolidone K60 (PVP) and hydroxypropyl cellulose (HPC 150–400 mPa s) were purchased from Tokyo Chemical Industry Co. Ltd. *p*-Phthalic acid (PTA) was purchased from Shanghai Aladdin Biochemical Technology Co. Ltd. Analytical grade chemicals were employed as received without further purification, and deionized (DI) water was used for all experiments.

### 2.2 The preparation of mesoporous black TiO_2_ hollow spheres

Colloidal silica particles were prepared *via* a modified Stöber method.^31^ Briefly, 3.5 mL of TEOS was mixed with a solution of ethanol (90 mL), water (17.5 mL) and ammonia (2.5 mL) under vigorous magnetic stirring (700 rpm, WH220-HT) for 4 h. The formed colloidal silica particles were collected by centrifuging twice with ethanol and re-dispersed in 40 mL of ethanol under ultrasonication (500 W, JY92-2D). The coating of the silica particles with a TiO_2_ layer was conducted according to a previous report^32^ with minor modification. Briefly, the silica particles (10 mL) were mixed with 0.4 mL of water in 90 mL ethanol, and 0.4 g of HPC was added to the mixture under vigorous stirring. 30 min later, 4 mL of TBOT in 20 mL ethanol was added dropwise by the peristaltic pump within 40 min, followed by refluxing for 120 min at 90 °C under magnetic stirring. The particles were collected by centrifuging twice with ethanol and once with water. Subsequently, the particles were re-dispersed in 60 mL of water to give a SiO_2_@TiO_2_ suspension followed by adding 0.8 g of PVP and stirring overnight for the absorption of PVP on the surfaces of TiO_2_, which facilitated subsequent SiO_2_ coating on the TiO_2_ surfaces. The particles treated with PVP were washed once with water and twice with ethanol by way of centrifugation, and then re-dispersed in a solution of 100 mL of ethanol, 17.5 mL of water, 3.44 mL of TEOS and 2.5 mL of ammonia under vigorous stirring. Four hours later, the suspension was centrifuged three times, and the precipitates were re-dispersed in ethanol and dried at 70 °C. Finally, the precipitates were calcined at 800 °C for 4 h to crystallize the amorphous TiO_2_ to anatase TiO_2_ grains.

The crystalline SiO_2_@TiO_2_@SiO_2_ (0.5 g) particles obtained from the above calcination were dispersed in 150 mL of water, and then 1.0 g of NaOH was added to the suspension to etch the outermost and innermost SiO_2_ of the particles at 90 °C for 5 h. This produced well-defined mesoporous TiO_2_ hollow spheres that were collected by centrifuging 5 times with ethanol to wash out the residuals, and then dried at 70 °C.

**Scheme 1:**
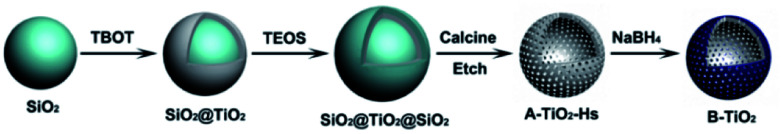
Schematic of the formation of black TiO_2_ hollow spheres.

The hollow TiO_2_ spheres were converted to black TiO_2_ using a modified NaBH_4_-reduction method.^24,25^ Briefly, 0.5 g of TiO_2_ hollow spheres were mixed with 0.5 g of NaBH_4_ (the ratio of TiO_2_ and NaBH_4_ was 1 : 1) and ground thoroughly. The mixture was put into a porcelain boat and then transferred to a tube furnace (CHY-1200), then heat-treated at 320 °C, 350 °C, 380 °C and 400 °C under the protection of Ar atmosphere for 40 min (the samples thus obtained were named T320, T350, T380, and T400, respectively). After the samples were cooled naturally, the formed black TiO_2_ hollow spheres were collected and then washed twice with ethanol and three times with water. The final precipitates were re-dispersed in water, separated and then dried at −52 °C using a vacuum freeze dryer (FD-1A-50). About 12 h later, the powders were collected. The whole synthesis procedure is shown in Scheme 1.

### 2.3 Characterization

The optical properties were detected by UV-Vis diffuse reflectance spectroscopy (UV-3600). The crystallinity was evaluated by X-ray diffraction (XRD) analysis (DX-2700, Dandong Fangyuan Corp., Liaoning, China). The morphology of the sample was characterized by transmission electron microscopy (TEM) (FEI Titan G2 60-300) and high-resolution transmission electron microscopy (HRTEM) (FEI Titan G2 60-300). X-ray photoelectron spectroscopy (XPS) (Thermo Fisher Scientific K-Alpha) was applied to analyze the valence state of the Ti element and the different binding states. Electron paramagnetic resonance (EPR) spectroscopy (Bruker E500) was employed to characterize the defects on the surfaces. An FL spectrophotometer (F-4600) was used to evaluate the concentration of ˙OH radicals using terephthalic acid photo-luminescence (TA-PL) with an excitation wavelength of 332 nm.

### 2.4 Photocatalytic degradation

The sample was used for the photocatalytic degradation of rhodamine B (RhB), for which a 300 W Xenon-lamp with a 400 nm cut-off filter was used as the visible light source. In a typical experiment, 30 mg of the sample was added to 250 mL of RhB aqueous solution (0.005 mM) and then dispersed under ultrasonication. A 60 min adsorption test under stirring in the dark was carried out to achieve an adsorption–desorption equilibrium before the visible light was turned on. Next, 4 mL of the suspension was sampled at a certain time interval and then filtered (1825-025) and centrifuged (8000 rpm for 2 min) for measuring the concentration of RhB using a UV 2000 spectrophotometer operating at 554.5 nm.

### 2.5 Photoelectrochemical experiments

Photocurrent measurements were done with a CHI 660E electrochemical workstation. The light source consisted of a 300 W Xe lamp with a cut off filter at 400 nm. Photocurrent measurements were performed with a CHI 660E electrochemical workstation. The light source consists of a 300 W Xe lamp with a cut off filter at 420 nm. The mixture was dip-coated onto a 1 cm × 2 cm FTO-glass to form a film as the working electrode. Pt foil, a saturated Hg/HgO electrode, and 0.5 M Na_2_SO_3_ were used as the counter electrode, reference electrode, and electrolyte, respectively. For electrochemical impedance spectroscopic (EIS) measurements, a three-electrode supercapacitor, composed of a Pt electrode and standard Hg/HgO reference electrode, was used; 3 M KOH was used as the electrolyte.

## 3. Results and discussion

### 3.1 The morphology of black hollow TiO_2_ photocatalysts

**Fig. 1:**
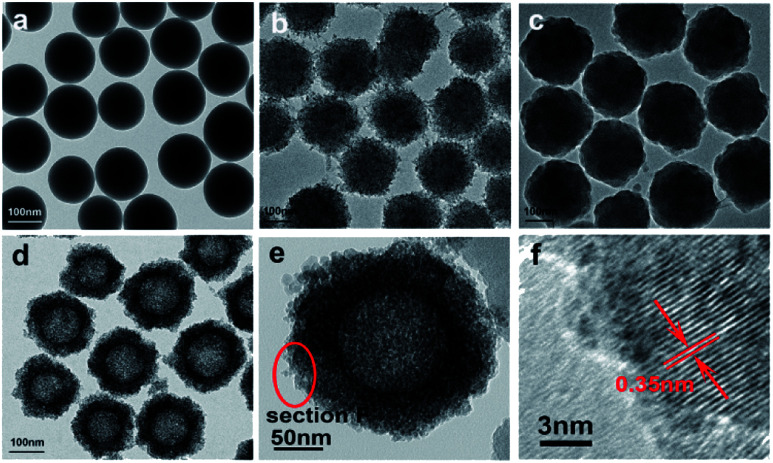
TEM of SiO_2_ spheres (a), SiO_2_@TiO_2_ spheres (b), SiO_2_@TiO_2_@SiO_2_ spheres (c), TiO_2_ hollow spheres (d), T350 (e), and the HRTEM of T350 (f).

The morphology of the prepared samples was characterized by TEM. Fig. 1a–c shows the SiO_2_ spheres, TiO_2_-coated SiO_2_ spheres, and SiO_2_–TiO_2_-coated SiO_2_ spheres, respectively. The SiO_2_ spheres exhibit good monodispersibility and smooth surface, with a uniform size of ∼150 nm (Fig. 1a). Fig. 1b shows that the TiO_2_-coated SiO_2_ spheres have a rough surface with a diameter of about 250 nm, demonstrating the successful coating of TiO_2_ outer layers on SiO_2_ spheres. As shown in Fig. 1c, the surfaces of the particles became smooth again after coating the TiO_2_ layer with SiO_2_, and these particles were larger than the pure SiO_2_ particles in Fig. 1a. Thus, one can conclude that the SiO_2_@TiO_2_@SiO_2_ structure was formed for the particles and they had good mono-dispersity. Furthermore, the mono-disperse TiO_2_ hollow sphere (Fig. 1d) formed by etching using NaOH had a size of ∼250 nm, which is consistent with the previous report.^32^ The TEM image of a black TiO_2_ particle (T350) formed by the reduction of the TiO_2_ hollow spheres using NaBH_4_ is shown in Fig. 1e; the particle still maintained the hollow sphere morphology. The HRTEM image of the particle (Fig. 1f) shows that there is an amorphous layer on the surface of the hollow TiO_2_ particle (Fig. 1e), which could be attributed to the formation of black TiO_2_.^1,15^ In contrast, the internal section has high crystallinity with the lattice spacing of ∼0.35 nm, which coincides with the (101) spacing of the anatase phase.

### 3.2 Structure and composition of hollow black TiO_2_ photocatalysts

**Fig. 2:**
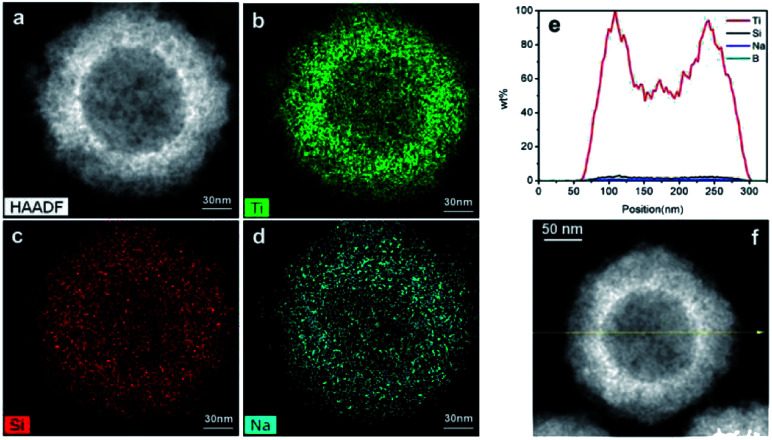
The map scanning (a–d) and the line scanning (e and f) of the black TiO_2_ hollow sphere (T350).

The elemental distribution was characterized by TEM line scanning and map scanning. Fig. 2a shows a typical black TiO_2_ hollow sphere and Fig. 2b–d shows the distribution of Ti, Si and Na elements, respectively. According to the mapping density of each element, the major component is Ti, followed by Si. As shown in Fig. 2d, elemental Na is still present, which could be due to the NaOH for etching or NaBH_4_ for Ti^4+^-reduction. The arrows in Fig. 2f shows the line scanning direction and the corresponding element content were shown in Fig. 2e. The Si content was very low, thus the vast majority of the SiO_2_ was removed by etching using NaOH. The content of element B was also very low, making it negligible in the black TiO_2_ hollow spheres, which is consistent with previous works.^24,25^

### 3.3 The effect of mesoporous hollow structure and catalyst concentration on photocatalytic performance

**Fig. 3:**
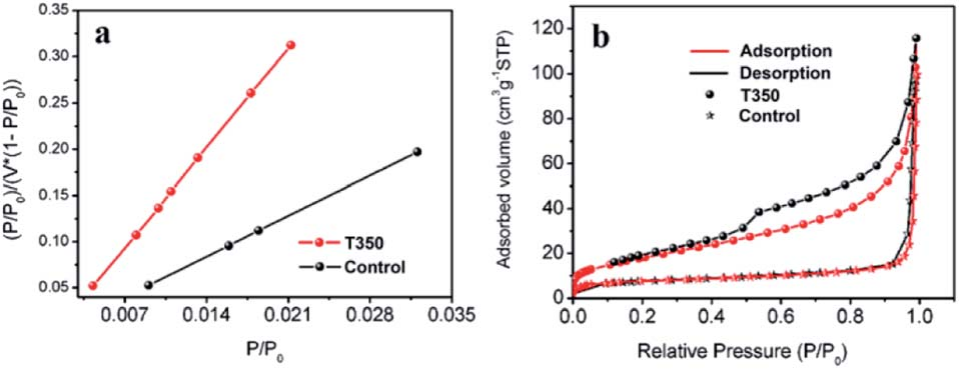
BET-adsorption-test results (a) and adsorption–desorption isotherms (b) of T350 and the control sample.

The effect of the specific surface area on the photocatalytic performance has been reported in many works.^33,34^ In our work, SiO_2_@black TiO_2_, the core–shell solid spheres, were chosen as the control group. The sample was obtained by reducing the SiO_2_@TiO_2_ spheres with NaBH_4_ at 350 °C, with the same reduction process as the black TiO_2_ hollow spheres (T350). The specific surface areas of the T350 and control samples were ∼66 m^2^ g^−1^ and ∼27 m^2^ g^−1^, respectively, similar to previous reports,^33,34^ as determined by the BET method (see Fig. 3a and Table S1†). The adsorption–desorption isotherm (Fig. 3b) was used to characterize the porosity of the samples. The formed hysteresis loop illustrates the richer pore structure on the surface of T350.^34^ Moreover, the distribution of the pores of the control sample and T350 are shown in Fig. S1.† There is a big difference between the control sample and T350 both in 2–10 nm and ∼120 nm, which means that a hierarchical porosity composed of mesopores (2–10 nm) connected with macropores (∼120 nm) was formed in the T350. Hierarchical porosity has been proven to favor multi-light scattering/reflection and facilitate fast mass transport, resulting in improved performance both in the enhanced harvesting of the exciting light and the improved photocatalytic activity.^35,36^ The large differences in specific surface area and pore structure make the hollow structure more suitable for photocatalysis because of more reaction sites and unique light absorption.

**Fig. 4:**
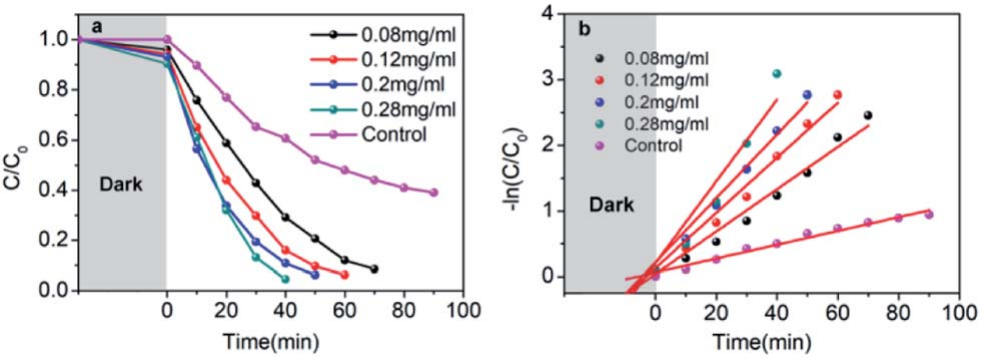
Photocatalytic degradation of RhB under visible-light irradiation of T350 with different concentrations, and the control sample (a); variations of −ln(*C*/*C*_0_) *versus* visible-light irradiation time (b).

The visible-light-driven photocatalysis of the black TiO_2_ hollow spheres (T350) and the control sample with different catalyst concentrations was investigated. RhB, a well-known organic pollutant, was chosen to test the photocatalytic performance of the samples. As shown in Fig. 4a, the black TiO_2_ hollow spheres exhibited improved photocatalytic properties. The catalyst concentration has an obvious influence on the catalytic efficiency, *i.e.*, catalytic efficiency increases as the catalyst concentration increases. When the catalyst content was increased to 0.28 mg mL^−1^, RhB was nearly fully catalyzed in 40 minutes, which is higher than the previous report.^37^ In contrast, the control group (0.28 mg mL^−1^) catalyzed only ∼50% in 90 minutes. The variation in the −ln(*C*/*C*_0_) *versus* the visible-light irradiation time is shown in Fig. 4b; it should be noted that the catalytic performance of T350 is ∼6 fold higher than that of the control group. The big difference in photocatalysis between the two cases can be ascribed to the hollow structure of the catalyst, as it can provide a larger specific surface area and more reactive sites.^34^

### 3.4 Structure and composition of hollow black TiO_2_ photocatalysts with different degrees of lattice disorder

**Fig. 5:**
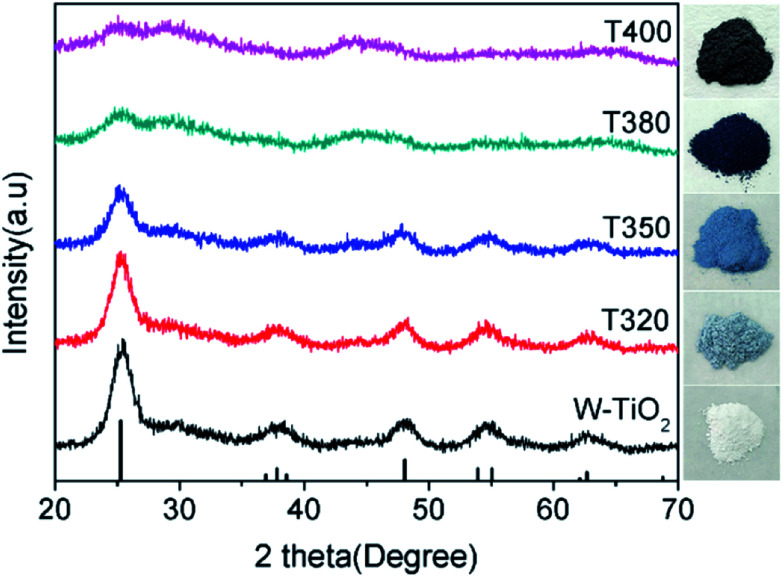
The XRD patterns of different samples and corresponding optical images.

To further elucidate the photocatalytic mechanism, a series of black TiO_2_ hollow spheres were reduced with NaBH_4_ at different temperatures to engineer different degrees of lattice disorder in the shells of the samples. The samples thus derived were examined using XRD for their crystalline structures, as shown in Fig. 5. The standard card (JCPDS no. 21-1272) of anatase TiO_2_ was used as a reference. As seen from Fig. 5, the white titanium dioxide (W-TiO_2_) exhibits high crystallinity, and its XRD pattern is consistent with the anatase phase of TiO_2_.^33^ Furthermore, the crystallinity of the samples became lower as the reduction temperature increased. Their optical images (right side of Fig. 5) show that the initial sample (W-TiO_2_) was white, whereas, with increasing reduction temperature, the samples became light blue, blue, black and dark brown. The change in color is an indication of the formation of black TiO_2_, due to the increased degree of surface lattice disorder and the decrease in Ti valence from Ti^4+^ to Ti^3+^ after treatment with NaBH_4_;^24^ the blacker the TiO_2_, the lower the ratio of O/Ti and the greater the lower valence state amount, similar to the previous report.^38^ This change is consistent with the evolution of XRD patterns in Fig. 5.

**Fig. 6:**
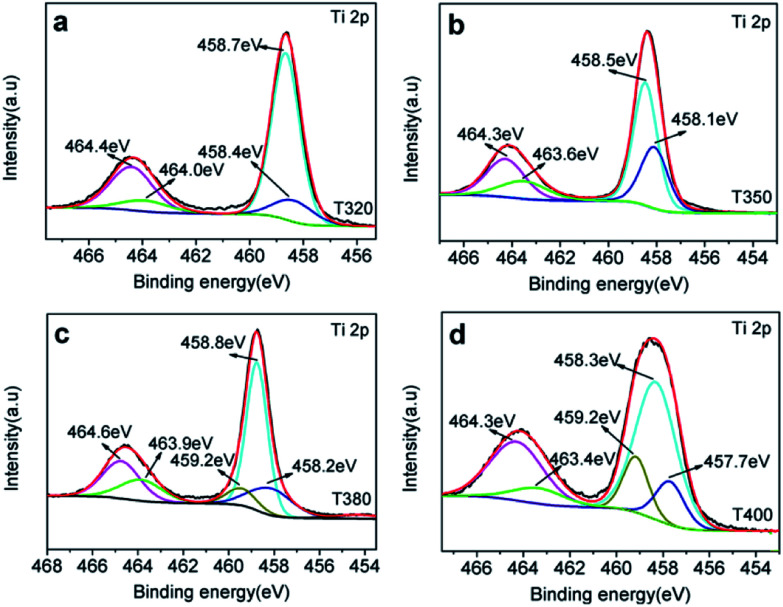
Ti 2p XPS spectra for (a) T320, (b) T350, (c) T380 and (d) T400.

XPS was used to detect the surface chemical binding of the powders reduced at different temperatures. The elements Ti, O, C and Si appeared in the full-scale XPS spectra, as shown in Fig. S2.† The appearance of C is due to the carbonation of organic molecules on the particle surfaces in heat treatment. Furthermore, the XPS peaks of Ti 2p in T320 can be fitted with four peaks centered at 458.4 eV, 458.7 eV, 464.0 eV, and 464.4 eV, and those in T350 with peaks centered at 458.1 eV, 458.5 eV, 463.6 eV, and 464.3 eV. These correspond to the binding energies of Ti^3+^ 2p_3/2_, Ti^4+^ 2p_3/2_, Ti^3+^ 2p_1/2_, and Ti^4+^ 2p_1/2_, respectively. Ti^3+^ emerged due to the effect of hydrogen reduction induced by the thermal decomposition of NaBH_4_.^24,25^ Besides, the ratios of Ti^3+^ to Ti^4+^ were about 32.5% (T320) and 51.4% (T350), as shown in Table 1, which were higher than most of the reports.^24,39,40^ Similarly, as shown in Fig. 6c and d, the peak of the T380 can fit to five peaks centered at 458.8 eV, 458.2 eV, 459.2 eV, 463.9 eV and 464.6 eV, and 458.3 eV, 457.7 eV, 459.2 eV, 463.4 eV and 464.3 eV in T400. The peaks centered at 458.8 eV (458.3 eV in T400), 458.2 eV (457.7 eV in T400), 463.9 eV (463.4 eV in T400), and 464.6 eV (464.3 eV in T400) correspond to the Ti^4+^ 2p_3/2_, Ti^3+^ 2p_3/2_, Ti^3+^ 2p_1/2_, and Ti^4+^ 2p_1/2_ respectively, and the peak centered at 459.2 eV belongs to Ti^2+^.^41^ It should be noted that Ti^3+^ emerges when the reduction temperature is 320 °C, as shown in Fig. 4a. The amount of Ti^3+^ increased when the reduction temperature increased to 350 °C. Ti^2+^ first appeared in the sample when the temperature increased further to 380 °C, and its content increased further when the reduction temperature was increased to 400 °C.^24^ The emergence of Ti^3+^ and Ti^2+^ can be due to the reduction of Ti^4+^, which could lead to the formation of oxygen vacancies. It indicated that the number of surface defects increased with increasing reduction temperature, as also suggested by the XRD data shown in Fig. 5, and by EPR data. The valence band (VB) XPS spectra of T350 and W-TiO_2_ are shown in Fig. S5.†

**Table 1:** Details of Ti 2p of the samples

Valence	Sample			
	T320	T350	T380	T400
Ti^4+^	75.5%	66%	62.2%	70.8%
Ti^3+^	24.50%	34%	27.3%	14.3%
Ti^2+^	—	—	10.5%	14.9%

**Fig. 7:**
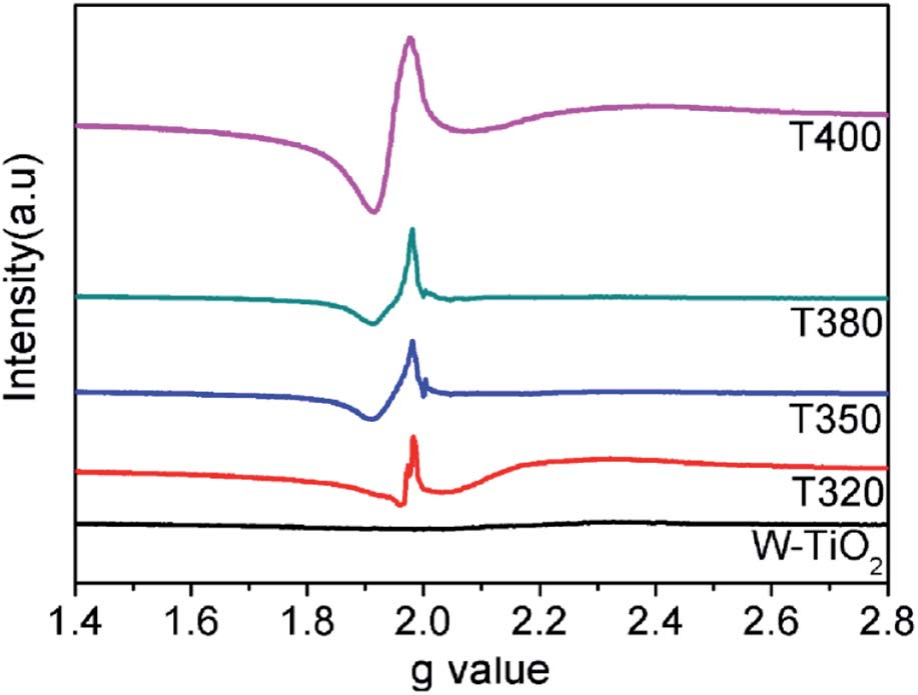
EPR spectra of the W-TiO_2_, T320, T350, T380 and T400.

EPR spectra were adopted to detect unpaired spins and magnetism on the surface of the sample, specifically the existence of Ti^3+^ ions and oxygen vacancies. As shown in Fig. 7, compared with the W-TiO_2_, the peaks centered at *g* = 1.945 emerged in the reduced samples. The intensities of these peaks increased as the reduction temperature increased, denoting that there were more defects formed on the surfaces. Theoretically, the peaks centered at *g* = 1.945 originated from the paramagnetic Ti^3+^ state, and the peaks at *g* = 2.002 in T350 and T380 correspond to unpaired electrons trapped by oxygen vacancies;^42^ both peaks are relevant to the photocatalysis. In T320 and T400, the signal from *g* = 2.002 is absent, which means that the peak at *g* = 1.945 can be composed of Ti^3+^ and some lower valence states, such as Ti^2+^,^24^ also seen in other materials.^43^ No EPR signal was detected in W-TiO_2_, indicating that the surface defects on black TiO_2_ particles were produced when the samples were treated with NaBH_4_, which corresponds to other reports.^24,25^

### 3.5 Optical properties of hollow black TiO_2_ photocatalysts

**Fig. 8:**
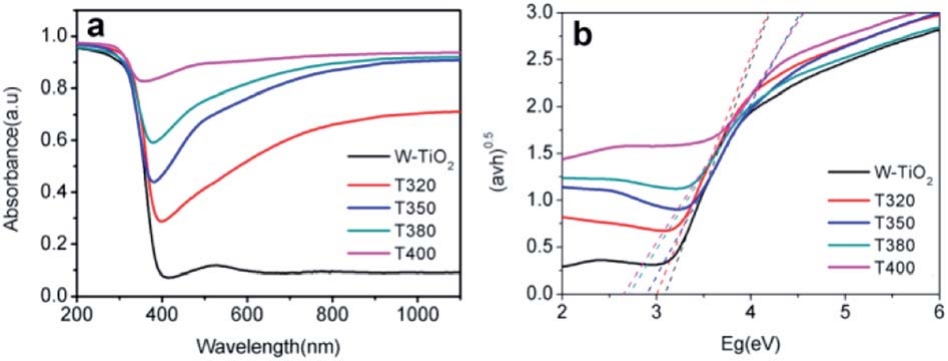
The UV-Vis absorption (a), and the corresponding value of band gap (b) of the samples.

The photo-absorption properties of the samples were characterized by UV-Vis, as shown in Fig. 8a. W-TiO_2_ possesses high absorption under ultraviolet irradiation, but weak absorption in visible light. In contrast, the reduced samples (T320, T350, T380, and T400) exhibited obvious absorption in visible light, and the intensity of the absorption increased as the reduction temperature gradually increased. The incredible changes in optical properties can be attributed to the treatment with NaBH_4_, which can produce Ti^3+^ and oxygen vacancies, similar to previous work.^24^ The values of the bandgap were also calculated according to the previous report^44^ and the corresponding results are shown in Fig. 8b. The as-prepared W-TiO_2_ has a bandgap of 3.07 eV, and the band gap values of T320, T350, T380 and T400 are 3.0 eV, 2.87 eV, 2.7 eV and 2.65 eV, respectively. It is commonly regarded that black TiO_2_ exhibits decreased band gap and improved visible-light absorption, which leads to enhanced and efficient visible light catalysis.^17^ We tried to verify this assertion using our photocatalytic experiments as described below.

### 3.6 Visible-light-driven catalytic activity and electrochemical properties

**Fig. 9:**
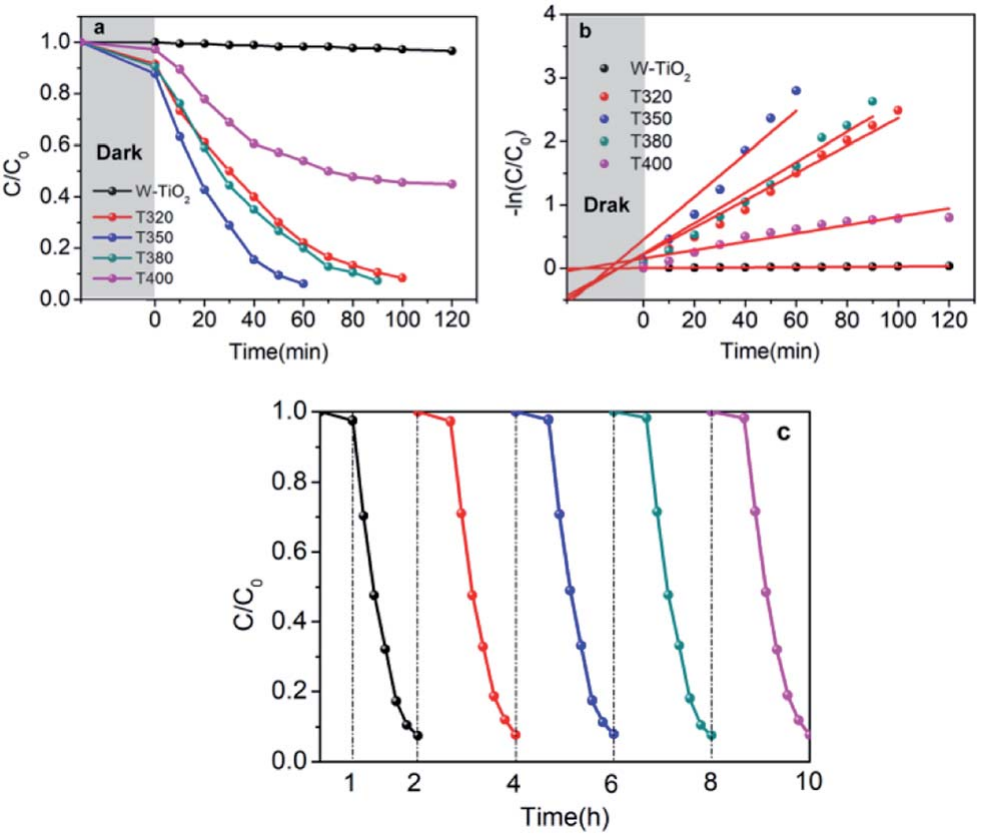
Photocatalytic degradation of RhB under visible-light irradiation (a), variations of −ln(*C*/*C*_0_) *versus* visible-light irradiation time (b) with a catalyst concentration of 0.12 mg mL^−1^. (c) Cycling tests of the visible-light-driven photocatalytic activity of T350.

**Fig. 10:**
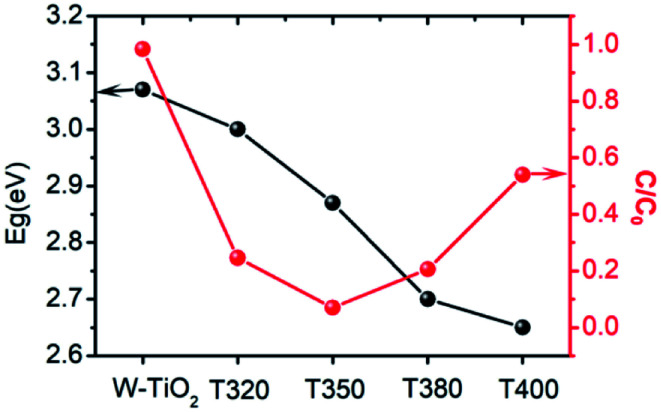
The change in band gap (black line) and catalytic efficiency in 1 h (red line) for different samples.

It can be seen from Fig. 9 that the samples obtained by reduction with NaBH_4_ at different temperatures have different visible-light photocatalytic effects. W-TiO_2_ was used as a control sample. As shown in Fig. 9a, using T320, T350, T380 and T400 as the photo-catalysts, the quantity of RhB was reduced by 75.5%, 93.1%, 79.6% and 46.2%, respectively, with a reaction time of 1 h. The variation of the −ln(*C*/*C*_0_) *versus* the visible-light irradiation time is shown in Fig. 9b, which suggests the degradation reaction is a first-order reaction. It is noted that the catalytic performance of T350 is ∼5 fold higher than that of T400, ∼1.4 fold that of T380 and ∼2 times that of T320. The cycling tests revealed that the T350 was very stable in five photocatalysis cycles shown in Fig. 9c. Generally, the higher visible light degradation performances in black TiO_2_ can attribute to the relatively high concentrations of defects and the smaller band gaps in the black TiO_2_ samples.^18^ As shown in Fig. 10, the values of the band gap decrease regularly with the reduction temperature. This tendency is consistent with that of the visible-light absorption properties. However, the catalytic efficiency is not proportional to the band gap, as observed in previous works.^24^ This demonstrates a contradiction between the visible-light degradation performance and band gap in black TiO_2_.

**Fig. 11:**
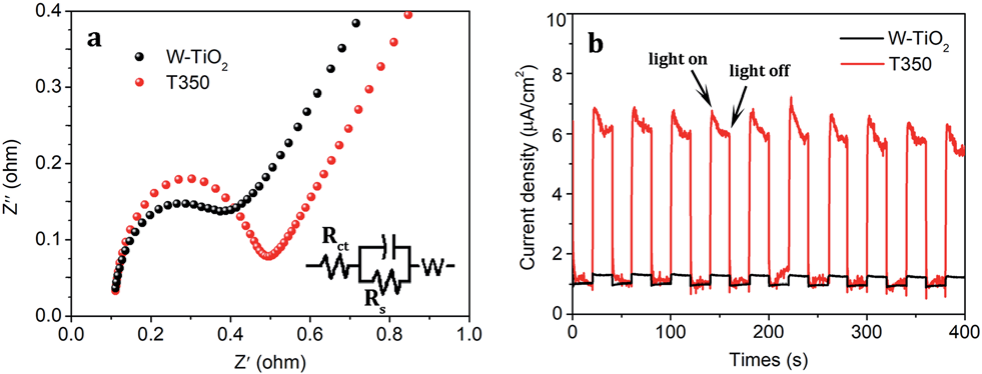
The (a) electrochemical impedance spectra and (b) photocurrent responses for W-TiO_2_ and T350.

Moreover, the EIS of the obtained samples is also characterized as shown in Fig. 11a. T350 has the smaller circular arc radius and the larger slope, which means that it has a higher separation efficiency of photoexcited electron–hole pairs than W-TiO_2_.^37^ The transient photocurrent responses of W-TiO_2_ and T350 are shown in Fig. 11b. The photocurrent response of T350 is about 5 times that of W-TiO_2_, which is consistent with the above properties, suggesting that photoexcited electron–hole pairs are highly separated.^35,45,46^ So the appearance of surface defects is considered to be the real factor that mainly affects photocatalysis.

### 3.7 Theoretical analysis and photocatalytic mechanism

According to the above description, we tried to identify the factors that predominantly determine the photocatalysis reaction. As shown in Fig. S3,† the concentration of surface OH species in T350 is lower than that in other samples. Nonetheless, it has the highest photocatalytic performance. Thus, surface OH species are not a determining factor. As shown in Fig. 8 and 9, the photocatalytic ability of a black TiO_2_ is not directly proportional to the visible light absorption (or the bandgap). Hence, the photo-absorption and band gap are also non-critical to the photocatalytic properties. As shown in Fig. 5, the amount of surface defects increases with the heat-treatment temperature,^24^ which would enhance the photocatalytic ability. However, the photocatalytic ability of black TiO_2_ is not proportional to the intensity of EPR either. The type of surface defects may also influence the photocatalysis by black TiO_2_. As shown in the XPS of Ti 2p (Fig. 4), the intensities of Ti^3+^ peaks increase from W-TiO_2_ (Fig. S4a†) to T350, but decrease from T350 to T400, whereas the Ti^2+^ species appear in T380 and its intensity increases with reduction temperature in T400. On correlating these facts with the photo-catalytic data in Fig. 7, we infer that Ti^3+^ plays a critical role in the catalysis when Ti^3+^ is present as in T320 and T350. However, when Ti^2+^ appears, such as in T380 and T400, it offsets the positive effect of Ti^3+^ and restrains the photocatalysis; thus, the relative concentrations of Ti^3+^ and Ti^2+^ species predominantly determine the catalytic capability of the black TiO_2_ under study.

**Scheme 2:**
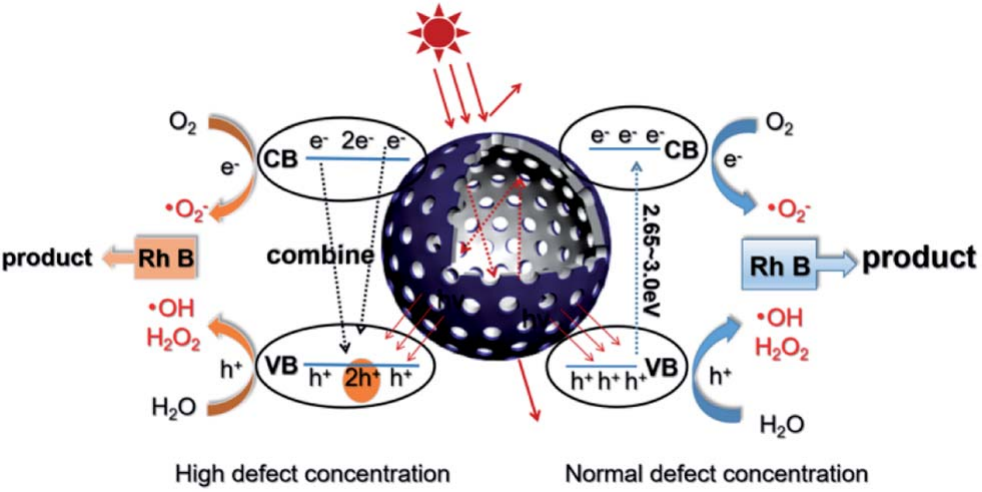
Schematic of the visible-light-driven photocatalytic mechanism and the light pathway in the hollow structure.

Based on the above discussions, a visible-light-driven photocatalytic mechanism can be proposed as shown in Scheme 2. The light pathways are shown in the particle. The efficiency of light utilization is greatly increased due to the multiple refractions of the hollow black TiO_2_ structure when compared to a solid sphere. The high specific surface area of the hollow spheres can provide more reaction sites, which also contributes to the enhanced photocatalysis.^33,34^

With a normal defect concentration as in the T320 or T350 samples, the photon-excited electrons (e^−^) are transferred from the VB to the CB when the photons (*hv* > *E*_g_) hit the surface of the sample, leaving holes (h^+^) in the VB and the formed electrons (e^−^) in the CB.^47^ The photon generated electrons (e^−^) can react with the oxygen dissolved in a solution to produce superoxide anion radicals (˙O_2_^−^), while the absorbed water can be oxidized by the holes (h^+^) to form hydroxide anions (˙OH) and hydrogen peroxide (H_2_O_2_) (see Fig. S6†). Both radicals (˙O_2_^−^ and ˙OH) can participate in the degradation of organic pollutants. The relevant reactions are shown below.^34,48^H_2_O + h^+^ → ˙OH + H^+^2˙OH + h^+^ → H_2_O_2_O_2_ + e^−^ → ˙O_2_^−^

With a high defect concentration as in the T380 or T400 samples, the defects can also act as charge recombination centers, as shown in Scheme 2. The excited electrons (e^−^) will fall back to the VB, which will decrease the photocatalytic ability of the sample. As stated above, we think defects associated with Ti^2+^ play the role of charge recombination centers,^49^ so the emergence of Ti^2+^ is harmful to photocatalysis. The formation process of Ti^2+^ is shown in Fig. S7.†

## 4. Conclusions

In this work, black TiO_2_ with mesoporous hollow shell structures were synthesized using the EISA method integrated with etching by NaOH and reduction by NaBH_4_. The unique structured black TiO_2_ exhibited significantly improved visible-light-driven photocatalytic properties, due to higher specific surface area, more reaction sites, and enhanced visible light absorption capability. Moreover, the black TiO_2_ hollow spheres with different lattice disorder-engineering degrees were designed by reducing the samples at 320 °C, 350 °C, 380 °C and 400 °C, respectively. It was found that the higher the reduction temperature, the greater the disorder in the black TiO_2_ samples, accompanied by higher visible light absorption. However, the photocatalytic efficiency changed with the processing temperature non-monotonically: it increased first and then decreased. This phenomenon can be explained by the varied functions of the surface defects at different concentrations in the black TiO_2_ during the catalytic process. Our work provides significant insight into the mechanism accounting for the high efficiency in the visible-light-driven catalysis by hollow black TiO_2_ spheres, which may guide the development of new applications for the newly fabricated black TiO_2_ materials.

## Conflicts of interest

There are no conflicts to declare.

## Supplementary Material

RA-009-C9RA08148H-s001

## References

[cit1] Chen X. B., Liu L., Peter Y. Y., Mao S. S. (2011). Science.

[cit2] Zhou W., Li W., Wang J. Q., Qu Y., Yang Y., Xie Y., Zhang K. F., Wang L., Fu H. G., Zhao D. Y. (2014). J. Am. Chem. Soc..

[cit3] Zhou T. H., Lv W., Li J., Zhou G. G., Zhao Y., Fan S. X., Liu B. L., Li B. H., Kang F. Y., Yang Q. H. (2017). Energy Environ. Sci..

[cit4] Zhang H., Lv X. J., Li Y. M., Wang Y., Li J. H. (2010). ACS Nano.

[cit5] Ullattil S. G., Periyat P. (2016). J. Mater. Chem. A.

[cit6] Banerjee S., Dionysiou D. D., Pillai S. C. (2015). Appl. Catal., B.

[cit7] Kale B. M., Wiener J., Militky J., Rwawiire S., Mishra R., Jacob K. I., Wang Y. J. (2016). Carbohydr. Polym..

[cit8] Wu B. H., Liu D. Y., Mubeen S., Chuong T. T., Moskovits M., Stucky G. D. (2016). J. Am. Chem. Soc..

[cit9] Sinhamahapatra A., Lee H. Y., Shen S. H., Mao S. S., Yu J. S. (2018). Appl. Catal., B.

[cit10] Warnan J., Willkomm J., Farré Y., Pellegrin Y., Boujtita M., Odobel F., Reisner E. (2019). Chem. Sci..

[cit11] Sundin E., Abrahamsson M. (2018). Chem. Commun..

[cit12] Chen X. B., Burda C. (2008). J. Am. Chem. Soc..

[cit13] Xu Y. F., Zhang C., Zhang L. X., Zhang X. H., Yao H. L., Shi J. L. (2016). Energy Environ. Sci..

[cit14] Seh Z. W., Liu S. H., Low M., Zhang S. Y., Liu Z. L., Mlayah A., Han M. Y. (2012). Adv. Mater..

[cit15] Lin T. Q., Yang C. G., Wang Z., Yin H., Lü X. J., Huang F. Q., Lin J. H., Xie X. M., Jiang M. H. (2014). Energy Environ. Sci..

[cit16] Fang Y., Sun M. G., Wang Y., Sun S. F., He J. (2016). Mater. Res. Bull..

[cit17] Wang Z., Yang C. Y., Lin T. Q., Yin H., Huang F. Q., Lin J. H., Xie X. M., Jiang M. H. (2013). Energy Environ. Sci..

[cit18] Wang Z., Yang C. Y., Lin T. Q., Yin H., Chen P., Wan D. Y., Xu F. F., Huang F. Q., Xie X. M., Jiang M. H. (2013). Adv. Funct. Mater..

[cit19] Yan Y., Han M., Konkin A., Koppe T., Wang D., Andreu T., Chen G., Vetter U., Morante J. R., Schaaf P. (2014). J. Mater. Chem. A.

[cit20] Dong J., Han J., Liu Y., Nakajima A., Matsushita S., Wei S., Gao W. (2014). ACS Appl. Mater. Interfaces.

[cit21] Li H., Chen Z., Tsang C. K., Li Z., Ran X., Lee C., Pan B. (2014). J. Mater. Chem. A.

[cit22] Shah M. W., Zhu Y., Fan X., Zhao J., Li Y., Asim S., Wang C. (2015). Sci. Rep..

[cit23] Shu G., Wang H., Zhao H. X., Zhang X. J. (2019). ACS Appl. Mater. Interfaces.

[cit24] Tan H. Q., Zhao Z., Niu M., Mao C. Y., Cao D. P., Cheng D. J., Feng P. Y., Sun Z. C. (2014). Nanoscale.

[cit25] Cao Y., Xin Z. P., Shen Y. C., Li Z. Z., Wu X. Y., Yan X., Zou J. L., Yan S. L., Zhou W. (2017). Chem. Eng. J..

[cit26] Zhang Y., Xing Z. P., Liu X. F., Li M., Zhu Q., Zhou W. (2016). ACS Appl. Mater. Interfaces.

[cit27] Xu W. J., Bai Y. C., Yin Y. D. (2018). Adv. Mater..

[cit28] Zhang W., He H., Tian Y., Lan K., Liu Q., Wang C. Y., Liu Y., Elzatahry A., Che R. C., Li W., Zhao D. Y. (2019). Chem. Sci..

[cit29] Wang X., Bai L. C., Liu H. Y., Yu X. F., Yin Y. D., Gao C. B. (2017). Adv. Funct. Mater..

[cit30] Liu N., Zhou X. M., Nguyen N. T., Peters K., Zoller F., Hwang I., Schmuki P. (2017). ChemSusChem.

[cit31] Stöber W., Fink A., Bohn E. (1968). J. Colloid Interface Sci..

[cit32] Joo J. B., Zhang Q., Dahl M., Lee I., Goebl J., Zaera F., Yin Y. D. (2012). Energy Environ. Sci..

[cit33] Joo Ji. B., Lee I., Dahl M., Moon G. D., Zaera F., Yin Y. D. (2013). Adv. Funct. Mater..

[cit34] Liu H. Y., Ma H., Joo J. B., Yin Y. D. (2016). Sci. China Mater..

[cit35] Fang B. Z., Bonakdarpour A., Reilly K., Xing Y. L., Taghipour F., Wilkinson D. P. (2014). ACS Appl. Mater. Interfaces.

[cit36] Fang B. Z., Kim J. H., Kim M. S., Yu J. S. (2013). Acc. Chem. Res..

[cit37] Cao Y., Xing Z. P., Hu M. Q., Li Z. Z., Wu X. Y., Zhao T. Y., Xiu Z. Y., Yan S. L., Zhou W. (2017). J. Catal..

[cit38] Ye M. M., Jia J., Wu Z. J., Qian C. X., Chen R., O'Brien P. G., Sun W., Dong Y. C., Ozin G. (2017). Adv. Energy Mater..

[cit39] Cui H. L., Zhao W., Yang C. Y., Yin H., Lin T. Q., Gua H., Huang F. Q. (2014). J. Mater. Chem. A.

[cit40] Chen X., Zhao D. X., Liu K. W., Wang C. R., Zhang Z. Z., Shen D. Z. (2015). ACS Appl. Mater. Interfaces.

[cit41] Hanawa T. (2011). J. Periodontal Implant Sci..

[cit42] Kumar C. P., Gopal N. O., Wang T. C., Wong M. S., Ke S. C. (2006). J. Phys. Chem. B.

[cit43] Su Y., Lang J., Li L., Guan K., Du C., Peng L., Han D., Wang X. J. (2013). J. Am. Chem. Soc..

[cit44] Roose B., Pathak S., Steiner U. (2015). Chem. Soc. Rev..

[cit45] Zhong W., Shen S., He M., Wang D., Wang Z., Lin Z., Yu J. (2019). Appl. Catal., B.

[cit46] Zhong W., Lin Z., Feng S., Wang D., Shen S., Zhang Q., Fang B. (2019). Nanoscale.

[cit47] Chen X. B., Li C., Grätzel M., Kosteckid R., Mao S. S. (2012). Chem. Soc. Rev..

[cit48] Nosaka Y., Nosaka A. Y. (2017). Chem. Rev..

[cit49] Su R., Tiruvalam R., He Q., Dimitratos N., Kesavan L., Hammond C., Hutchings G. J., Besenbacher F. (2012). ACS Nano.

