# Supplementary material for: Mesoporous hollow black TiO2 with controlled lattice disorder degrees for highly efficient visible-light-driven photocatalysis
Source: RSC Adv. 2019 Nov 12;9(63):36907–14. doi: 10.1039/c9ra08148h (PMC9075178; doi:10.1039/c9ra08148h)
Supplement: RA-009-C9RA08148H-s001 [file RA-009-C9RA08148H-s001.pdf]

Electronic Supplementary Material (ESI) for RSC Advances.
This journal is © The Royal Society of Chemistry 2019

Supporting information for: Mesoporous hollow black TiO_2 with controlled lattice disorder degrees for highly efficient visible-light-driven photocatalysis

Xiongrui Jiang,[a] Zhiyao Yan,[a] Jing Zhang,[b] Junzheng Gao,[a] Wanxia Huang[a], Qiwu Shi*[a],*

Hengzhong Zhang[c]

[a] College of Materials Science and Engineering, Sichuan University, Chengdu 610065, Sichuan, China

[b] College of Architecture and Environment, Sichuan University, Chengdu 610065, Sichuan, China

[c] Center for High Pressure Science and Technology Advance Research, Shanghai 201203, China

The BET details of Control sample and T350.

Table S1 The basic information of BET of Control sample and T350

	k	b	Vm [ml]	C	BET [m^2/g]
Control sample	0.159447	0.00064	6.2466	250.143	27.188
T350	0.064788	0.00097	15.2079	67.98	66.192

Where the k represent the slope, b is Y-intercept, Vm is Single layer saturation adsorption capacity, C is the sorption constant and the BET represent the BET Surface Area.

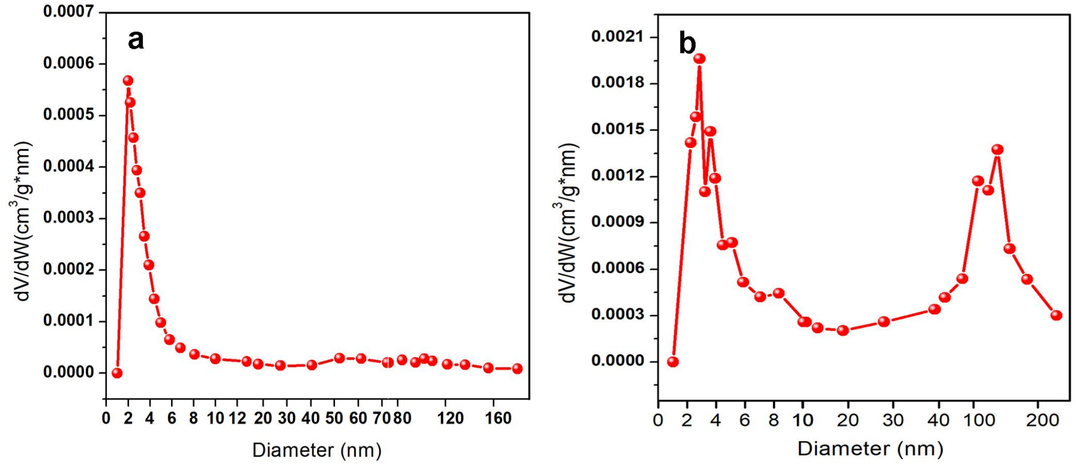

Fig. S1 The pores distribution of Control sample (a) and T350 (b).

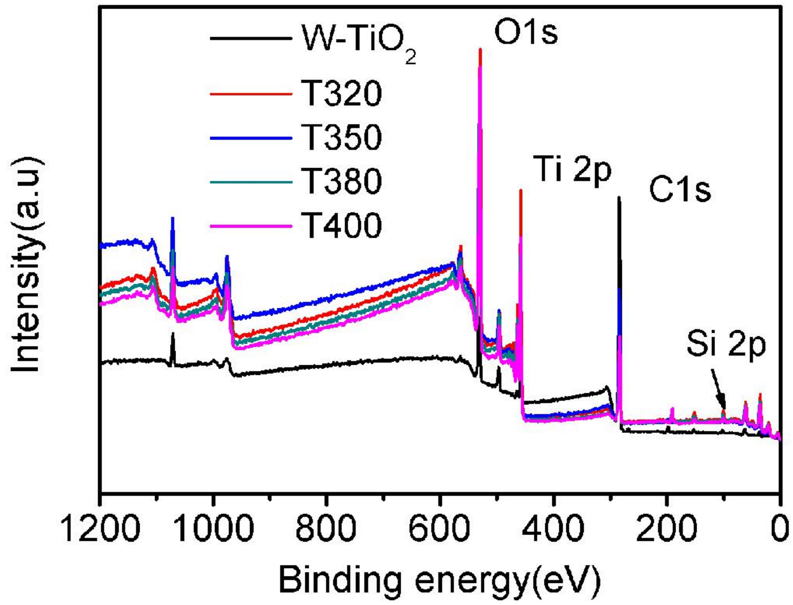

Fig. S2 The full XPS spectra of samples.

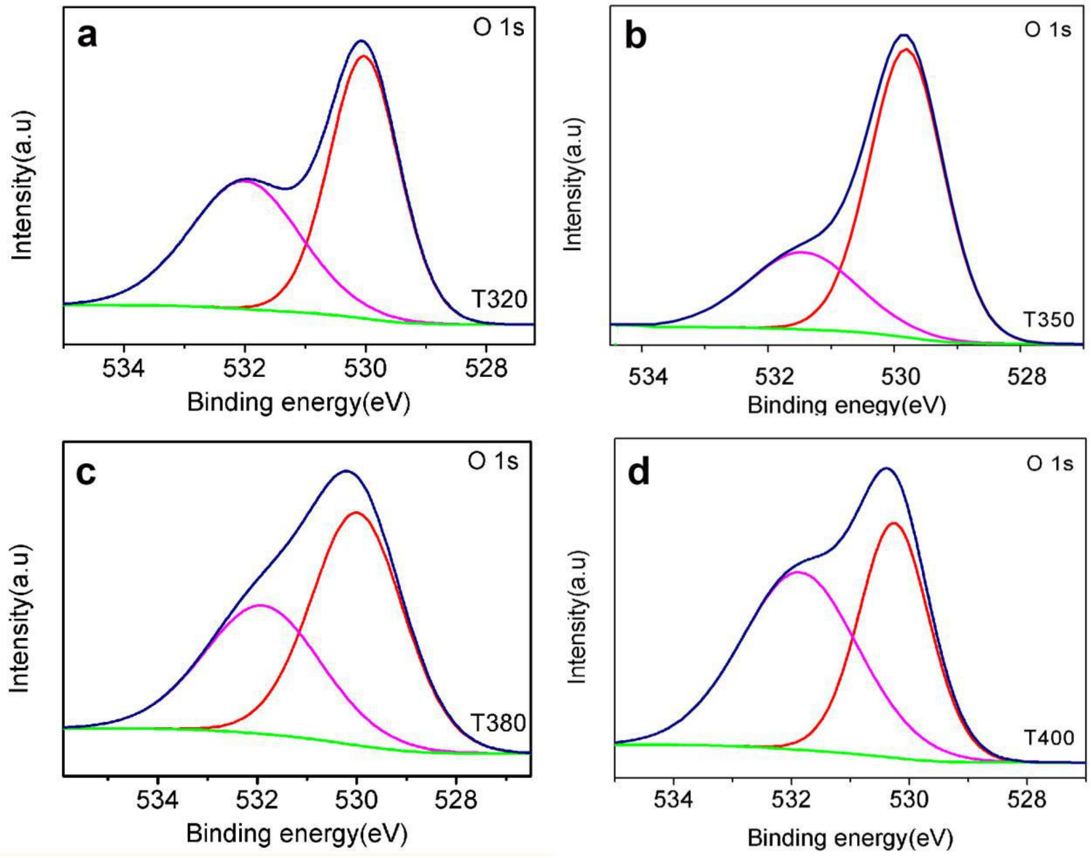

Fig. S3 The XPS spectra of O 1s. (a)T320, (b)T350, (c)T380 and (d)T400.

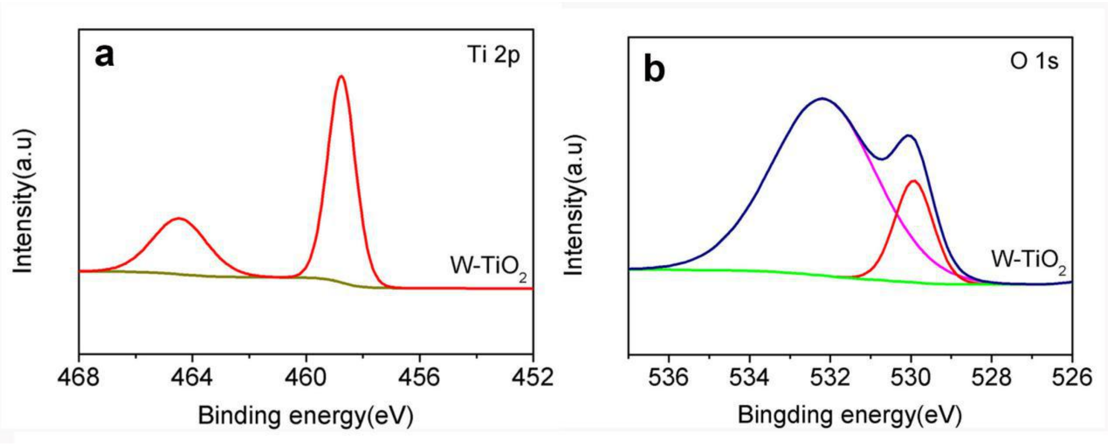

Fig. S4 The XPS spectra of W-TiO_2. (a)Ti 2p, (b)O 1s.

The valence band (VB) XPS spectra of W-TiO_2 and T350.

The VB XPS spectra were used to determine the VB changes in the W-TiO_2 and T350 samples. The results are shown in Fig. S5a. The W-TiO_2 displayed band edge at 2.43 eV,

corresponding to the valence band maximum (VBM). As its band gap is 3.02 eV (Fig. 8b), its conduction band minimum (CBM) equals 2.42–3.02 = -0.59 eV, as shown in Fig. S5b. After treatment with $NaBH_4$ in the T350 sample, the VBM changed to 1.62 eV. Hence, its CBM became 2.16–2.87 (band gap) = -1.25 eV. As the VBM of T350 is smaller than that of W-TiO_2, a lower photon energy is required to excited the electrons from the VB to the CB, moving the useful light spectrum by the former to the longer wavelength regime.

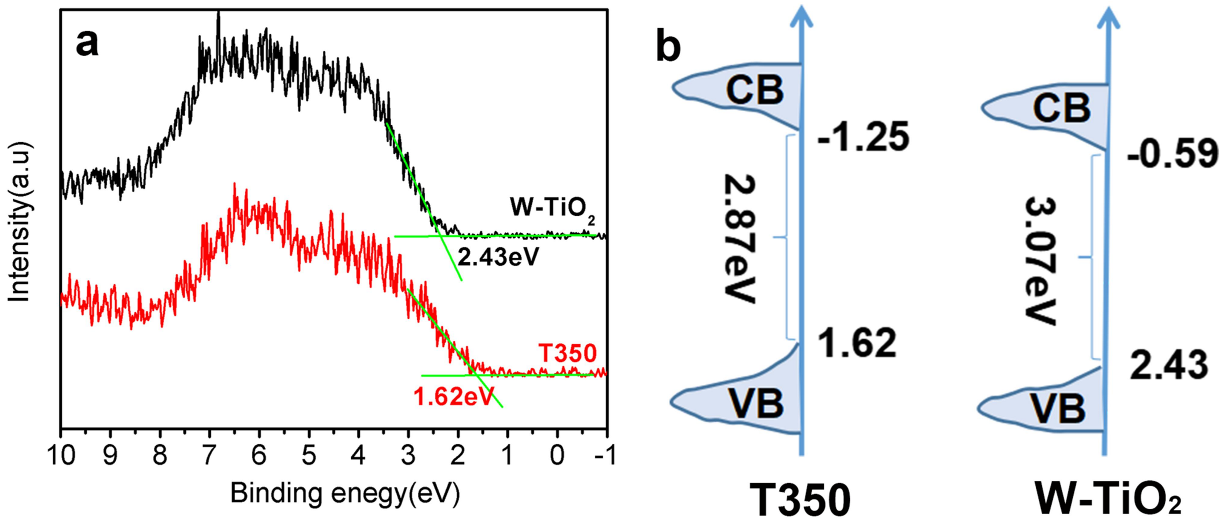

Fig. S5. Valence band (VB) spectra (a) and schematic electronic structures (b) for W-TiO_2 and T350.

The photoluminescence of W-TiO_2 and T350.

The photoluminescence (PL) emission spectra was used to understand the behavior of light-generated electrons and holes. [1,2] Fig. S4 reveals the fluorescence intensity of W-TiO_2 and T350 under Xenon lamp irradiation with a 400 nm cut-off filter for 1 h (30 mg of a catalyst was added into 250 ml of a 0.5 mM PTA aqueous solution with a NaOH

concentration of 0.2 mM). It can be concluded that T350 produced more •OH radicals than $W\text{-}TiO_2$, which is consistent with the result of Rh B degradation experiment.

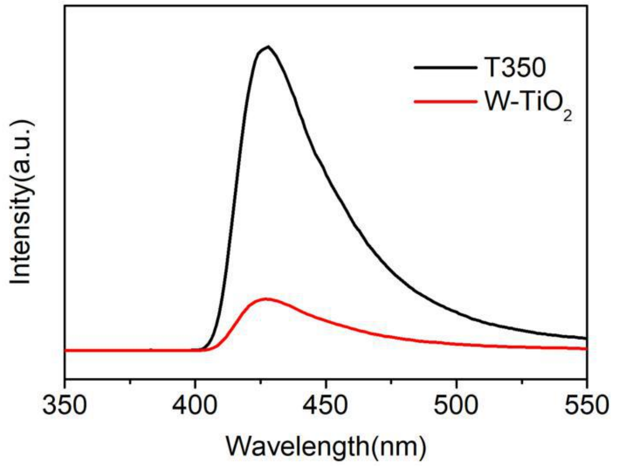

Fig. S6 Fluorescence spectra of $W\text{-}TiO_2$ and T350 under visible light by using 300W Xeon-lamp with a 400 nm cut-off filter for 1 h in 0.5 mM terephthalic acid.

The formation of Ti^{2+}.

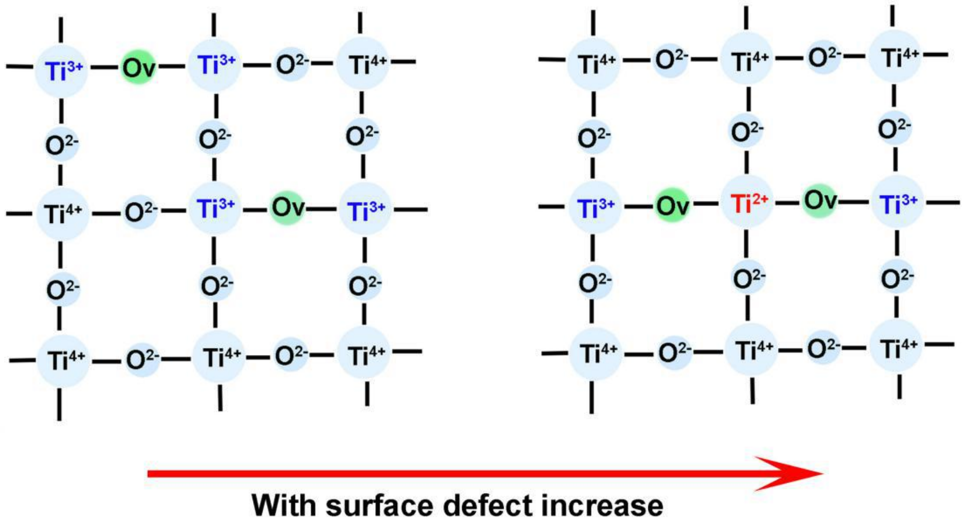

Fig. S7 Proposed sample lattice structure of TiO_2 with different defect concentration.[3]

Reference

1 J. C. Yu, G. J. Yu, W. K. Ho, Z. T. Jiang, L. Zhang, *Chem. Mater.* **2002**. 14, 3808–3816 .

2 Q. Xiang, K. Lv, J. Yu, *Applied Catalysis B: Environmental,* **2010**, 96, 557–564 .

3 M. J. Xu, Y. Chen, J. T. Qin, Y. W. Feng, W. Li, W. Chen, H. X. Li and Z. F. Bian, Environ. Sci. Technol. **2018**, 52, 13879−13886.
